# Kinematic and spatiotemporal assessment of habituation to treadmill walking in Labrador retrievers

**DOI:** 10.1186/s13028-016-0265-9

**Published:** 2016-12-28

**Authors:** Pia Gustås, Kjerstin Pettersson, Sofia Honkavaara, Anne-Sofie Lagerstedt, Anna Byström

**Affiliations:** 1 Department of Clinical Sciences, Faculty of Veterinary Medicine and Animal Husbandry, Swedish University of Agricultural Sciences, Box 7054, 750 07 Uppsala, Sweden; 2 University Animal Hospital, Swedish University of Agricultural Sciences, Box 7040, 750 07 Uppsala, Sweden; 3 Department of Anatomy, Physiology and Biochemistry, Faculty of Veterinary Medicine and Animal Husbandry, Swedish University of Agricultural Sciences, Box 7011, 750 07 Uppsala, Sweden; 4 Evidensia Specialistdjursjukhuset Helsingborg, Bergavägen 3, 250 23 Helsingborg, Sweden

**Keywords:** Canine, Kinematics, Locomotion, Orthopaedics, Rehabilitation

## Abstract

**Background:**

This study investigated differences in kinematic and spatiotemporal variables in Labrador retrievers during introduction to treadmill walking, with the aim to determine the time required for them to become habituated. Twenty-five healthy, treadmill-naive Labrador retrievers participated in the study. The total angular displacement of the carpus, elbow, tarsus and stifle, as well as stride time and stance duration were calculated from the 3-D tracking of skin mounted reflective markers recorded with 6 infrared light emitting video cameras at 240 Hz. The measurements were done at two walking speeds, 0.78 and 0.96 m/s, in six sessions on the treadmill during two consecutive days.

**Results:**

With a 1–2 min acclimatization period following each treadmill speed change, mean values of the study variables were significantly different from the last training session mainly in the first session on the first day. However, between-stride variability was significantly larger for at least one variable even in the fourth session for the slower walking speed, and in the fifth session for the higher walking speed.

**Conclusions:**

The results show the importance of proper pre-training of dogs in locomotion studies at walk using a treadmill, and the need to consider not only variable mean values but also between-stride variability, in order to ensure that dogs are sufficiently accustomed to allow collection of reliable data.

## Background

A gait is formed through complex interactions between the musculoskeletal and the central and peripheral nervous system, where ambulation requires constant adaptation to both intrinsic and extrinsic factors. Orthopedic and other conditions that affect the function of either the nervous or the musculoskeletal system typically disrupt these interactions and thus lead to gait disturbances. To describe any such changes in a dog’s locomotor function, a systematic approach with good repeatability of the selected gait variables is required. Because of movement variability, i.e. the inherent variation between strides present even within a stable gait pattern, it is necessary to record data from several strides to obtain a representative mean value [1–5]. Further, the influence of extrinsic factors, which may increase this variability, should be minimized during data collection, to obtain reproducible results from a minimum number of strides [6–10].

In order to record several strides from a stable gait, a treadmill has been found to be a useful tool, in human [11] and equine [12] as well as in canine [13] locomotion research. The advantage of a treadmill is that it provides a well-controlled setting with constant speed. Some studies in dogs have used a treadmill to record walking kinematics in healthy dogs [14, 15], as well as in dogs with different orthopaedic conditions [16–22].

The rationale behind using a treadmill is based on the assumption that its use induces a stable, repeatable gait. This is well established for humans, horses and dogs. On the other hand, for subjects unfamiliar with the treadmill it is known that their gait is characterised by both divergent mean values and increased between-stride variability [11, 23–25]. To obtain reliable data, it is therefore important to give new subjects enough time to become accustomed to a treadmill [11, 23, 26, 27]. This presents a challenge for measurements of dogs at walk because no studies have described the process of adaptation in dogs. Treadmill training of dogs in trot has been described [24, 25, 28–30] but because a study in horses showed that longer training time was needed to achieve habituation in walk compared to trot [23], recommendations for trotting dogs cannot safely be applied to walk.

This study aimed to describe the pattern of gait adaptation in naïve Labrador retrievers when trained to walk on a treadmill, and to determine the training time needed for them to become accustomed to treadmill walking. It was hypothesized that being unfamiliar with treadmill walking would change kinematic and spatiotemporal variables and increase the between-stride variability, and that these changes would decrease with time and evolve into a regular, repeatable walking pattern in six sessions of training during two days, similar to observations in our previous study on treadmill training of naïve Labrador retrievers in trot [25].

## Methods

Data were collected in connection with our earlier study on treadmill habituation in trot [25]. The experimental setup has been described in detail earlier [25] and is therefore only summarized here.

### Animals

Twenty-five healthy privately owned Labrador Retrievers (22–34 kg) aged 22–36 months were used. All dogs passed an orthopaedic examination on entering the study. No dog had previous experience of treadmill exercise. Informed consent was obtained from the owners, in accordance with the ethical guidelines for the study, approved by the Uppsala Animal Ethics Committee, Sweden and Swedish Board of Agriculture (No. C32/7).

### Data collection

A rubber-belt treadmill (Rodby, Vänge, Sweden) with a 1.44 × 0.54-m running area was used. Dogs were always handled by the same person, held on a leash in the same manner. A motion analysis system with six infrared light emitting video cameras (Proreflex, Qualisys, Gothenburg, Sweden) was used to record three-dimensional kinematic data (240 Hz) using the proprietary camera software (Qualisys Track Manager, Qualisys, Gothenburg, Sweden).

A total of 26 spherical reflective markers, diameter 12 mm, were attached to each dog’s skin by the same person, with double-sided adhesive tape and glue over specific anatomical landmarks. Marker positions for the forelimbs were as follows: the distal end of the fifth metacarpal bone, the lateral styloid process, the lateral epicondyle of the humerus and the acromion; and for the hind limbs: the distal end of the fifth metatarsal bone, the lateral malleolus, the lateral epicondyle of the femur and the greater trochanter of the femur.

Each dog performed six sessions on the treadmill, three on each day on two consecutive days. Between sessions dogs rested for 30 min. Each session lasted 8–10 min and was divided into four consecutive 2-min trials at different treadmill speeds. In session 1 the dogs only walked: in trial 1 at 0.78 m/s, in trial 2 at 0.96 m/s, in trial 3 at 0.78 m/s and in trial 4 at 0.96 m/s. For session 2 to 6, dogs first walked and then trotted: trial 1 was walk at 0.78 m/s; trial 2 was walk at 0.96 m/s; trial 3 was trot at 1.81 m/s and trial 4 was trot at 2.06 m/s. The walking speeds were aimed to be a slow walk and a normal-to-fast walk. Kinematic data were recorded for 10 s every 30 s, i.e. four 10-s motion data capture periods were recorded at each speed. The first motion capture period started as soon as the treadmill reached the predetermined speed for the specific trial. For each walking speed, the 10-s motion capture periods from each session were termed 1.1–1.4 and 1.5–1.8 (for session 1), 2.1–2.4 (for session 2) and so on up to 6.1–6.4 (for session 6). The number of strides recorded in each motion capture period ranged from 8 to 10.

The data from each motion capture period was divided into strides using a custom written program (MathWorks Inc, Natick, Mass). First contact and toe-off for each limb were determined from the vertical and horizontal velocity of the marker of the distal fifth metacarpal or metatarsal bone, respectively, in a semi-automatic procedure adapted from a method previously used for equine treadmill trotting [31].

To quantify the basic gait characteristics a selection of spatiotemporal and kinematic variables were calculated: Spatiotemporal variables were stride time (time between two consecutive first contacts of the left hind limb), and stance time (time between first contact and toe-off) and relative stance time (stance time divided by stride time) for each limb. The kinematic variables calculated were total angular displacement, i.e. the difference between stride maximum and minimum joint angles, for the carpal, elbow, tarsal and stifle joints, respectively.

### Statistical methods

For all variables, mean and standard deviation (SD) were calculated for each dog and 10-s motion capture period. Group mean and a 95% confidence interval (CI) were then calculated for each motion capture period, and these values were plotted for each walking speed and variable to visualize overlaps and differences between the consecutive motion capture periods.

For each speed and variable, the distribution of dog mean values across motion data capture periods were evaluated by the study of overlaps between the 95% CIs, and with paired *t* test of the respective periods with that of the last motion capture period (6.4). Further, differences in the distribution of SDs (used as a measure of between-stride variability) between the last motion capture period and the previous periods were compared using a Wilcoxon signed rank test (a non-parametric test was chosen because the SDs were not normally distributed).

## Results

**Fig. 1:**
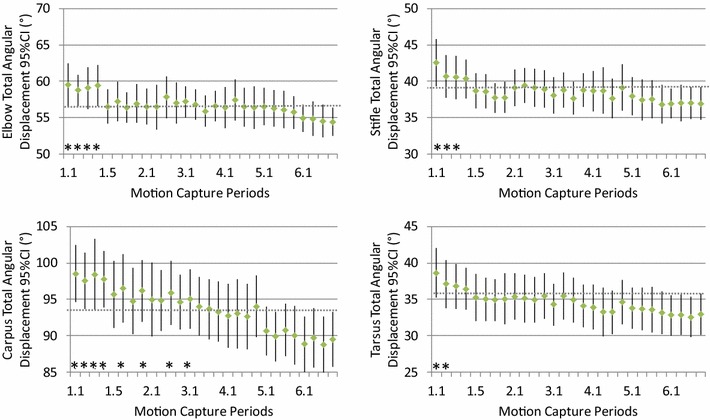
Group mean values (*dot*) and 95% confidence intervals (*error bars*) for the total angular displacement of the elbow, carpus, stifle and tarsus in dogs (n = 25) walking on a treadmill at 0.96 m/s. Motion capture periods deviating significantly (*P* < 0.05) from the final motion capture period (6.4) are marked “*”

**Fig. 2:**
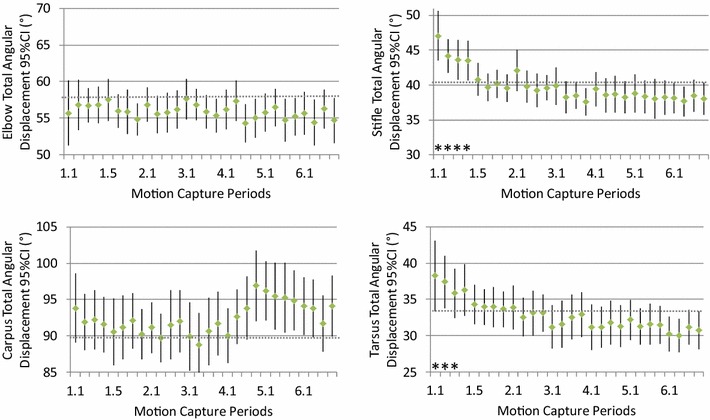
Group mean values (*dot*) and 95% confidence intervals (*error bars*) for the total angular displacement of the elbow, carpus, stifle and tarsus in dogs (n = 25) walking on a treadmill at 0.78 m/s. Motion capture periods deviating significantly (*P* < 0.05) from the final motion capture period (6.4) are marked “*”

Total angular displacements remained largely unchanged during the study (Figs. 1, 2). Significant differences compared to the final motion capture period (6.4) were observed during the first 4 min of walking on the treadmill, i.e. the trial 1 at each speed in the first session. During motion capture periods 1.1–1.4 the total angular displacements were significantly larger for all evaluated joints, except for the elbow and carpus at the slower walking speed (0.78 m/s). At the faster walking speed (0.96 m/s) the carpus total angular displacement also tended to be larger during the second session and in the first motion capture period of the third session.

**Fig. 3:**
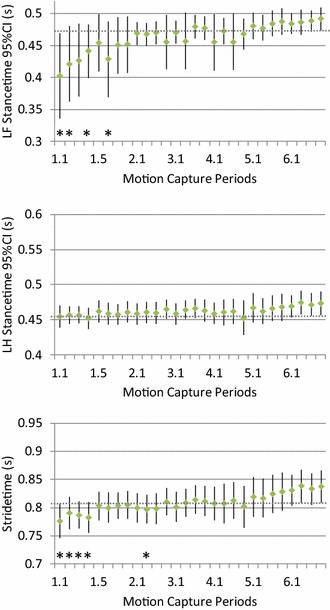
Group mean values (*dot*) and the 95% confidence intervals (*error bars*) for the stride time, left forelimb (LF) and left hind limb (LH) stance in dogs (n = 25) walking on a treadmill at 0.96 m/s. Motion capture periods deviating significantly (*P* < 0.05) from the final motion capture period (6.4) are marked “*”

**Fig. 4:**
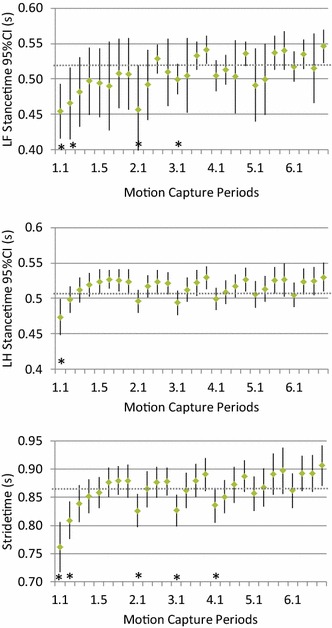
Group mean values (*dot*) and the 95% confidence intervals (*error bars*) for the stride time, left forelimb (LF) and left hind limb (LH) stance in dogs (n = 25) walking on a treadmill at 0.78 m/s. Motion capture periods deviating significantly (*P* < 0.05) from the final motion capture period (6.4) are marked “*”

The spatiotemporal variables followed a similar pattern as the angular displacements (Figs. 3, 4); significant differences compared to the final motion capture period (6.4) were observed in motion capture periods 1.1–1.4 at each speed, and were more prominent at the slower walking speed (0.78 m/s). Both stride and stance times were shorter during the first session on the treadmill, and combined with the increased angular displacements this gave the walk an abnormal visual appearance. At 0.95 m/s stride time was shorter also in the second session, while at the slower walking speed (0.78 m/s) both stride and stance times tended to be shorter during the first two motion capture periods in each of the following sessions (Fig. 4), i.e. during the dogs’ first minute on the treadmill after a rest period.

The intra-individual between-stride variability at the walk, measured as the intra-individual SD for each variable, changed for longer than the stride mean values (Table 1). At the higher walking speed (0.96 m/s) the SDs for stride time and elbow and carpal total angular displacements were significantly larger compared with the last motion capture period (6.4) even at the end of the 5th session (5.4). At the slower walking speed (0.78 m/s) the intra-individual variability decreased faster during the habituation: already in the second motion capture period of the fourth session (4.2) the SDs did not differ significantly from the SDs of the last motion capture period (6.4) for any variable, with stride time and stifle total angular displacement being the last variables to stabilize. Further, if the first two motion capture periods in each session were excluded as time to acclimate after a rest period or change in speed, then the second motion capture period of the third session (3.2) became the last session at slow walk (0.78 m/s) for which the SDs for any variable were significantly larger compared with the final motion capture period (6.4). In contrast, at the higher walking speed (0.96 m/s) exclusion of the first minute in each session only slightly influenced the results: the only change in time of stabilization was for the total angular displacement of the stifle (Table 2).

**Table 1:** Spatiotemporal variables

Speed	0.78 m/s				0.96 m/s			
*P*-level	<0.001	<0.01	<0.05	<0.05^#^	<0.001	<0.01	<0.05	<0.05^#^
Stride time	1.2	3.1	4.1	2.4	2.1	4.2	5.4	5.4
Stance time LF	1.1	1.2	1.3	1.3	1.2	1.8	2.4	2.4
Stance time LH	1.3	2.1	3.1	2.4	2.1	4.1	4.4	4.4
Relative stance time LF	n/a	n/a	n/a	n/a	n/a	n/a	n/a	n/a
Relative stance time LH	1.2	1.5	2.1	1.7	1.1	1.1	1.3	1.3

Between-stride variability decrease for stride and stance times during treadmill training of dogs (n = 25) in walk at 0.78 and 0.96 m/s, respectively. Table values represent the last 10-s motion capture period during which the intra-individual SDs were significantly higher compared with the final motion capture period (6.4), determined from a paired Wilcoxon sign rank test

^#^ First two motion capture periods in each session excluded

**Table 2:** Angular displacements

Speed	0.78 m/s				0.96 m/s			
*P*-level	<0.001	<0.01	<0.05	<0.05^#^	<0.001	<0.01	<0.05	<0.05^#^
Elbow	1.5	2.1	2.3	2.3	3.3	5.2	5.4	5.4
Carpus	n/a	n/a	n/a	n/a	5.1	5.1	5.4	5.4
Stifle	1.2	3.1	4.1	2.4	1.8	3.2	5.1	4.3
Tarsus	1.4	2.1	3.1	1.8	3.2	5.1	5.1	4.4

Between-stride variability decrease for the stride total angular displacement of the elbow, carpus, stifle and tarsus during treadmill training of dogs (n = 25) in walk at 0.78 and 0.96 m/s, respectively. Table values represent the last 10-s motion capture period during which the intra-individual SDs were significantly higher compared with the final motion capture period (6.4), determined from a paired Wilcoxon sign rank test

^#^ First two motion capture periods in each session excluded

## Discussion

Dogs on their first encounter with the treadmill showed a clearly aberrant gait pattern: during the first 4 min the stride mean angular displacements of the joints were significantly larger, mean stance and stride times were significantly shorter, and the between-stride variability was significantly higher compared with in the final session. This is in accordance with findings in previous studies on walking humans [26, 32] and horses [23] and trotting dogs [24, 25]; it has been found that the gait of subjects unfamiliar with the treadmill is characterized by an altered movement pattern and increased between-stride variability. Further, in the current study the initial divergences in mean values were larger in walk compared with the same dogs in trot [25]. This could be related to the study design: all dogs performed their very first training session on the treadmill in walk only and trot was not included until the second session. However, a study in horses also found that individuals needed longer training time to become fully accustomed to treadmill walking compared with treadmill trotting [23]. Based on the latter finding, it can be speculated whether the longer training time for walk might be related to the differences in limb coordination and pattern of mechanical work that exist between walk and trot because these differences are common to both dogs and horses [33, 34].

As the training of the dogs progressed, the mean total angular displacement and stride and stance times soon stabilised at values not significantly different from those obtained in the final session (Figs. 1, 2, 3 and 4). This indicates that a marked gait adaptation occurred already during the initial walking sessions. However, the between-stride variability of the same variables, quantified as the intra-individual standard deviation over all strides recorded during a 10-s motion capture period, continued to decrease over a longer time before non-significant levels were reached (Tables 1, 2). This is in accordance with previous studies in humans [11, 35] and horses [23] and in trotting dogs [25]. Thus, the general pattern for treadmill gait adaptation appears to be that a longer training time is required to reduce variability of the gait variables, compared with the time needed for the gait to be just on average similar to the subject’s gait when fully accustomed [23, 25, 35]. This stresses the need to not only assess mean values but also variability to determine whether a subject is fully accustomed to treadmill locomotion at a particular gait.

When comparing the literature on treadmill training of humans and horses at walk, it can be noted that the criteria for when a subject is considered sufficiently accustomed to treadmill locomotion varies somewhat between studies. In both an early human study and an equine study, full habituation was considered to have occurred when a consistent gait pattern was re-established in less than 1 or 2 min when returning to the treadmill for a new session [11, 23]. However, in later human studies, the subjects were considered to be sufficiently accustomed when a stable gait pattern was observed after 5 to 10 min [26, 27, 32, 35]. The current study therefore assessed how time to habituation was affected if a 1-min acclimatization period was allowed for at the beginning of each session. Dogs were fully habituated to treadmill walking in the fifth session at the slower speed (0.78 m/s) and in the sixth session at the higher speed (0.96 m/s). In these respective sessions none of the measured variables, neither mean values nor SDs, differed significantly from the last motion capture period (6.4), not even during the first minute of the session (motion capture periods 5.1–2 and 6.1–2, respectively). If the first minute of treadmill walking at each speed (motion capture period X.1 and X.2) are excluded and referred to as acclimatization period, then habituation was achieved already in the third session at the slower speed (0.78 m/s). At the higher speed (0.96 m/s), however, time to habituation did not differ depending on whether a 1-min acclimatization was allowed for or not; at this speed significant differences compared with the last motion capture period (6.4) were found even in the last motion capture period of the fifth session. A change in speed within the same gait thus seems to be equivalent to a break because the slower walk always preceded the faster walk in each session and yet the dogs required more training time to habituate in the faster walking speed. One reason habituation was achieved with less training time at the slower speed may be that this speed better matched the dogs’ preferred walking speed, and that it was therefore relatively more demanding for the dogs to adapt to treadmill walking at the faster speed.

An important aim of the current study was to evaluate the training time needed for dogs to get accustomed to treadmill walking. Our results suggest that five 8- to 10-min sessions during two consecutive days is sufficient to ensure full habituation to treadmill walking, and that two training sessions on a single day may be sufficient if the dogs are allowed a 1-min acclimatisation period after a break or change in speed. However, some limitations must be considered if our findings are to be applied as guidelines for treadmill training of dogs in future studies. One limitation is the duration of the training period. Two days of training is still a relatively short period and possibly additional more subtle adaptations may have continued if the training had continued. The time to full habituation would then have been longer, if variable values had been compared to values obtained after e.g. 2 weeks of daily training. Another limitation is the observed variation in required training time between speeds, and also between individuals. The fact that between-stride variability at group level did not decrease significantly after the fifth session does not imply that every single individual had reached minimum between-stride variability at that point. Finally, the systematic difference between the two speeds, as discussed above, indicates that the choice of walking speed influences the training time needed. Because of these limitations it is advisable that a margin of safety is applied if training time is scheduled based on the results of the current study to ensure that a particular dog is fully accustomed to treadmill walking in an arbitrary speed.

## Conclusions

Being unfamiliar with treadmill walking significantly affects kinematic and spatiotemporal variables, initially both measurement mean values and between-stride variability. Longer treadmill training is needed to achieve low-normal between-stride variability, compared with the time needed to obtain stable measurement mean values. To obtain reliable data for gait analysis, it must therefore be ensured that participating dogs are fully accustomed to treadmill walking. The current study provides some guidance to the training time needed, but variation between individuals and the difference found between a slower and faster walking speed indicates that generalising the guidance to other groups of walking dogs should be done with caution.
